# Task-dependent reorganization of functional connectivity networks during visual semantic decision making

**DOI:** 10.1002/brb3.286

**Published:** 2014-09-23

**Authors:** Matthew N DeSalvo, Linda Douw, Shigetoshi Takaya, Hesheng Liu, Steven M Stufflebeam

**Affiliations:** 1 Athinoula A. Martinos Center for Biomedical ImagingCharlestown, Massachusetts; 2 Massachusetts General HospitalBoston, Massachusetts

**Keywords:** Connectomics, default mode network, functional magnetic resonance imaging, graph theory, language, memory, resting state

## Abstract

**Introduction:**

Functional MRI is widely used to study task-related changes in neuronal activity as well as resting-state functional connectivity. In this study, we explore task-related changes in functional connectivity networks using fMRI. Dynamic connectivity may represent a new measure of neural network robustness that would impact both clinical and research efforts. However, prior studies of task-related changes in functional connectivity have shown apparently conflicting results, leading to several competing hypotheses regarding the relationship between task-related and resting-state brain networks.

**Methods:**

We used a graph theory-based network approach to compare functional connectivity in healthy subjects between the resting state and when performing a clinically used semantic decision task. We analyzed fMRI data from 21 healthy, right-handed subjects.

**Results:**

While three nonoverlapping, highly intraconnected functional modules were observed in the resting state, an additional language-related module emerged during the semantic decision task. Both overall and within-module connectivity were greater in default mode network (DMN) and classical language areas during semantic decision making compared to rest, while between-module connectivity was diffusely greater at rest, revealing a more widely distributed pattern of functional connectivity at rest.

**Conclusions:**

The results of this study suggest that there are differences in network topology between resting and task states. Specifically, semantic decision making is associated with a reduction in distributed connectivity through hub areas of the DMN as well as an increase in connectivity within both default and language networks.

## Introduction

Functional magnetic resonance imaging (fMRI) has been established as an important tool to noninvasively study both resting and task- or stimulus-related neural activity in both clinical and research settings. Early fMRI research focused on the measurement of patterns of activation and deactivation among brain regions in task performance paradigms. This research led to the development of fMRI-based clinical techniques to create presurgical maps of eloquent cortex. More recently, the study of functional connectivity has emerged as an active area of fMRI research. In this approach, regions are interpreted as being functionally connected when correlations among low-frequency blood oxygen level dependent (BOLD) signals exceed a threshold (Biswal et al. 1995). This has led to the characterization of a variety of functional neural networks that have been implicated in both normal cognitive processes and disease states. However, because most of these experiments have been conducted during the “resting-state” in which no task or stimulus is present, there is a limited understanding of how cognitive tasks modulate functional connectivity networks.

While several studies have explored task-related changes in functional connectivity, the results of these studies have been inconsistent (Esposito et al. 2006, 2009; Hampson et al. 2006; Calhoun et al. 2008; Buckner et al. 2009; Marrelec and Fransson 2011; Newton et al. 2011; Gordon et al. 2012; Li et al. 2012). Their interpretation has also been complicated by methodological inconsistencies wherein typically a small subset of user-defined brain regions is studied using either a seed-based approach or visual inspection of independent component analysis, both of which can introduce bias (Daubechies et al. 2009; Cole et al. 2010).

The variable results of prior studies have led to competing hypotheses regarding the relationship between task-related and resting-state functional networks. Some studies have shown similar organization between task-related and resting-state networks (Biswal et al. 1995; Arfanakis et al. 2000; Buckner et al. 2009; Smith et al. 2009) as well as the ability to predict task-related activations from intrinsic BOLD fluctuations (Fox et al. 2006; Raichle 2011); based on these studies, it has been proposed that resting-state networks act as “priors” for task networks. However, other studies have demonstrated reorganization of functional networks during task performance (Esposito et al. 2006, 2009; Calhoun et al. 2008; Marrelec and Fransson 2011; Newton et al. 2011), providing evidence that resting-state networks represent inactive or idle states of a dynamic system.

To complement the aforementioned approaches to studying connectivity, the fields of graph theory and connectomics have emerged, in which brain regions are represented as nodes of a graph, with edges that describe interregional connectivity derived from either structural or functional data (Rubinov and Sporns 2010). Graph theory can be used to quantify and compare properties of these complex networks across different experimental groups or settings. In this approach, connectivity among widespread brain regions can be studied with fewer a priori assumptions. In contrast to prior studies, in this experiment, we employed a data-driven graph-based approach to study task-related changes in functional connectivity across the entire cortex.

Evaluating changes in functional connectivity among brain regions in an unbiased, data-driven way is of critical importance as more experiments emerge that relate connectivity to both physiological and pathological processes. For example, from prior studies, specific resting-state networks have been consistently described including the highly studied default mode network (DMN), a set of brain regions that are activated and correlated during task-free introspection (Raichle et al. 2001; Greicius et al. 2003). Functional connectivity in this network has been shown to be increased during cognitive tasks such as working memory performance (Yakushev et al. 2013) and dysfunctional in neuropsychiatric conditions such as autism, schizophrenia, and dementia (Buckner et al. 2008). The regions most robustly identified include the medial prefrontal cortices (MPFC), inferior parietal lobules (IPL), precuneus, and retrosplenial/posterior cingulate (Rs/PCC) cortices as the DMN. While the DMN has been proposed to underlie a dominant mode of brain function based on unconstrained spontaneous physiological activity (Raichle and Gusnard 2005), it has also been implicated in conscious, task-unrelated cognitive processing of semantic thoughts (Binder et al. 1999). Understanding how task-related activity modulates functional connectivity within the DMN may provide key insight into the role of this network as critical connectivity hub in both health and disease (Buckner et al. 2009). Furthermore, a dynamic functional connectivity approach may provide further insight into task-related changes in connectivity among other networks throughout the brain that may allow clinicians to better utilize task-based and resting-state fMRI for presurgical planning.

**Figure 1:**
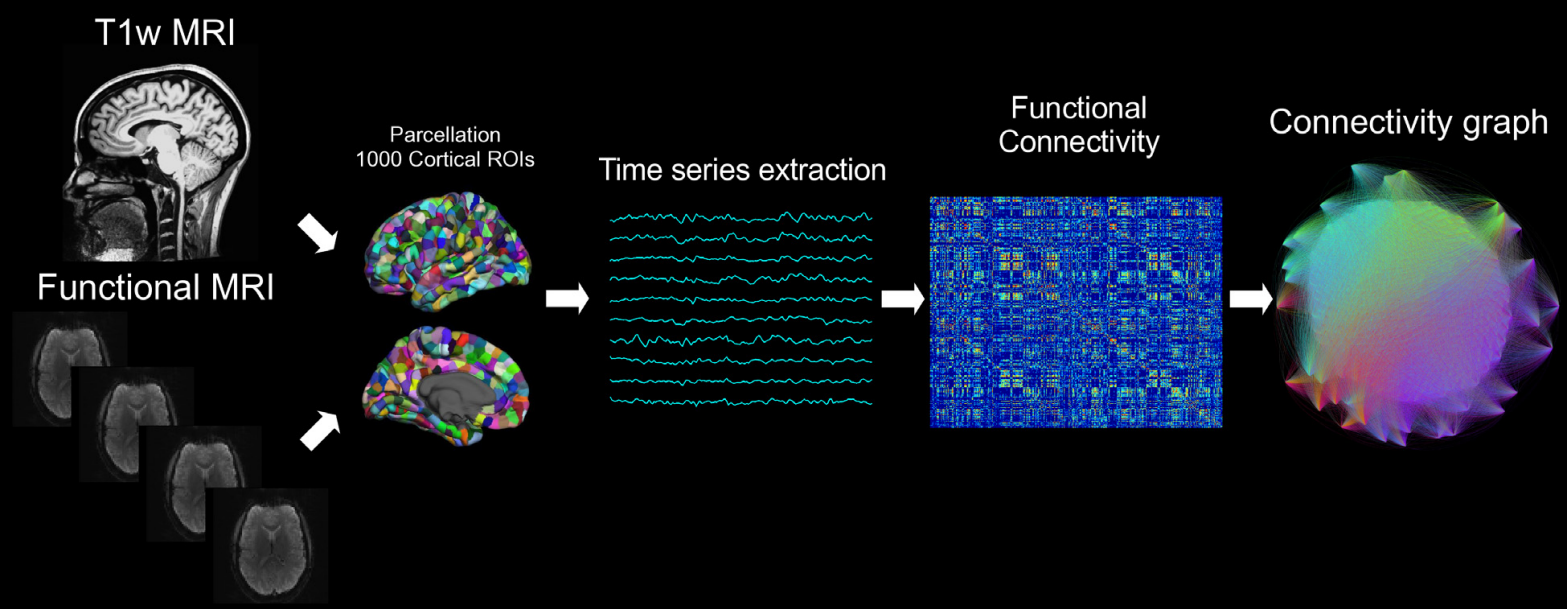
Schematic of analysis methodology. This flowchart illustrates the steps of this functional connectivity analysis. MPRAGE volumes were segmented using FreeSurfer and further parcellated into 1000 cortical ROIs. Time series extraction was performed after fMRI preprocessing, and the functional connectivity between each pair of cortical ROIs was calculated using Pearson's correlation coefficient. These sets of correlations, which defined an adjacency matrix for each run, were thresholded to retain the greatest 40% of correlations, and graph theory was applied to quantify network connectivity properties.

The principal aim of this study was to implement a graph-based network approach to analyze differences in functional connectivity among brain regions in healthy subjects during a cognitive task compared to rest. While most prior studies have primarily employed an n-back working memory task to explore task-dependent changes in functional connectivity, in this experiment we used a semantic decision task where a subject must mentally categorize abstract and concrete words (Demb et al. 1995; Gabrieli et al. 1996; Wagner et al. 1998). Because this is a complex task that involves a wide range of language processing and semantic memory, we hypothesized that both classical language areas such as the inferior frontal gyrus, posterior superior temporal gyrus, and angular gyrus areas as well as DMN areas, including the posterior cingulate cortex, precuneus, inferior parietal lobules, and medial orbitofrontal cortices, would exhibit different connectivity patterns when performing this task compared to rest. In brief, we calculated correlations in low-frequency BOLD signals across the entire cortex of healthy subjects, both while at rest and during a semantic decision task. We then used graph theory to quantify connectivity among regions, and statistically compared these values between rest and task runs (Fig.1).

## Methods

### Subjects

Data from 21 healthy right-handed subjects (ages 20–55 (mean ± SD = 26.7 ± 9.0), six men and 15 women) who were recruited from the community were retrospectively analyzed. Subjects were native English speakers with normal or corrected-to-normal vision, no history of neuropsychiatric conditions, and no active use of psychoactive medications. This study was approved by our institutional review board and all subjects gave signed informed consent.

### Data acquisition

Magnetic resonance imaging was performed on a 3T TimTrio system (Siemens, Erlangen, Germany) with a vendor-produced 32-channel head coil. High-resolution T1-weighted MP-RAGE sequences were acquired with TR = 2530 ms, TE = 1.74 ms, flip angle = 7° resulting in a matrix of 256 × 256 × 176 isotropic 1 mm voxels. Resting-state functional data were acquired using a gradient-echo echo-planar pulse sequence sensitive to BOLD contrast with TR = 5000 ms, TE = 30 ms, flip angle = 90°, 55 axial slices of 128 × 112 voxels at 2 mm isotropic resolution resulting in 76 frames over 380 sec. Task runs were acquired with TR = 2000 ms, TE = 30 ms, flip angle = 90°, 33 axial 3.6 mm thick slices of 64 × 64 voxels at 3 mm × 3 mm in-plane resolution resulting in 116 frames over 232 sec.

### Experimental paradigm

Each subject underwent 2 resting-state runs during which they passively fixated on a visual cross-hair centered on a screen. Patients were instructed to stay awake and remain as still as possible without any additional task instruction. Each subject underwent 3 task runs in which they decided whether centrally presented visual words represented abstract or concrete entities (Demb et al. 1995). Participants were instructed to respond promptly and accurately with a right-hand keypress. Task runs were organized in alternating blocks of 28 sec of rest and 36 sec of task with a new word presented every 3 sec, for a total of seven blocks. Stimuli were projected onto a screen positioned at the head of the magnet bore. Resting-state data were consistently acquired after task runs, and the stimulus paradigm was the same across all subjects.

### MRI preprocessing

Using FreeSurfer version 5.1 (http://surfer.nmr.mgh.harvard.edu) (RRID:nif-0000-00304) and the Lausanne 2008 parcellation provided by the Connectome Mapper (www.connectomics.org) (RRID:nlx_153920), cortical surface representations of MPRAGE volumes were divided into 1000 regions of interest (ROIs) that spanned the entire cortex.

MRI preprocessing procedures for functional data were based on those applied by Buckner et al. (2009) and adapted to a surface-based approach. These procedures included removal of the first four volumes to allow for T1-equilibration effects, compensation for slice-dependent time shifts, motion correction, and projection to FreeSurfer fsaverage5 surface. Motion correction results were inspected to ensure that each run had <1 mm translation and <1° rotation in any direction. Motion-corrected fMRI data were registered to the FreeSurfer processed MPRAGE volume. Temporal filtering removed constant and linear trends over each run while retaining frequencies below 0.08 Hz. Several sources of spurious variance were regressed: six parameters of rigid body head motion, whole-brain average signal, signal averaged over the lateral ventricles, and signal averaged over the deep cerebral white matter. Finally, processed fMRI data were averaged across each ROI to yield 1000 time series.

### Functional connectivity

Functional connectivity between pairwise ROIs was calculated using Pearson's correlation coefficient. Because removal of global signal causes a negative shift in the distribution of correlation coefficients introducing spurious anticorrelations (Keller et al. 2013), only correlations above the 60th percentile (top 40%) were retained, removing all negative correlations.

### Graph theory

This set of thresholded pairwise correlations was used to define a 1000 × 1000 weighted, undirected adjacency matrix for each run. Network properties were calculated using the Brain Connectivity Toolbox (https://sites.google.com/site/bctnet/) (RRID:nlx_143925) and customized MATLAB R2012b scripts (Mathworks, Inc., Natick, MA). For each ROI, we calculated the following network parameters:

*Degree*: the number of nonzero connections involving a ROI.

**Figure 2:**
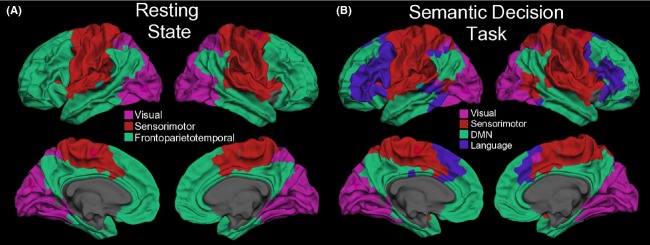
Task-related differences in modular structure. Surface representation at the gray–white matter junction of the modules that emerged using Newman's spectral algorithm on an across-subjects average connectivity adjacency matrix for both resting state (A) and task (B) runs. Colors are arbitrary.

*Modularity*: the extent to which a network can be divided into nonoverlapping sets of ROIs defined by maximizing within-module connectivity while minimizing between-module connectivity. We applied Newman's spectral algorithm (Newman 2006) to define modules in our data. Within-module connectivity was calculated using the z-score of within-module degree for each ROI, while between-module connectivity was calculated using the participation coefficient (Rubinov and Sporns 2010). To view the anatomical distribution of these modules, we displayed them as color-coded regions on cortical surface templates (Fig.2).

### Statistics

Connectivity measures were averaged across the three task runs and two rest runs for each subject, and were compared using pairwise Student's *t*-tests with correction for multiple comparisons using false discovery rate at *q* < 0.05.

## Results

We observed widespread task-dependent changes in connectivity throughout the brain. For brevity, we will refer to MPFC, IPL, precuneus cortex, and Rs/PCC as the DMN. Task-related changes in graph theory measures are summarized in Table 1.

**Table 1:** Summary of task-related differences in graph theory measures.

	Task	Rest
Overall connectivity (degree)	DMN Bilateral posterior insulae Right anterior temporal cortex	Bilateral sensorimotor cortices Bilateral SFG Bilateral inferior lateral frontoinsular cortex Left superior temporal cortex
Within-module connectivity (z-score of within-module degree)	Bilateral precunei Bilateral insulae Bilateral SFG Left IFG	Bilateral sensorimotor cortices Bilateral visual cortices
Between-module connectivity (participation coefficient)	Left dorsomedial angular gyrus	Diffusely, especially bilateral inferior frontal and insular cortices

Regions are listed in the column during which they had greater connectivity (*q* < 0.05). DMN, default mode network; SFG, superior frontal gyrus; IFG, inferior frontal gyrus.

### Modularity

Analysis of resting-state networks revealed three distinct modules: an occipital visual module, a sensorimotor module, and a large frontoparietotemporal module that included the DMN (Fig.2A). By contrast, although similar visual and sensorimotor modules were identified in task networks, the large frontoparietotemporal module seen in the resting-state was split into two modules: one consisting of portions of bilateral inferior frontal gyri and presupplementary motor areas (pre-SMA) as well as left posterior superior temporal gyri and another module that included DMN areas (Fig.2B).

### Overall connectivity

**Figure 3:**
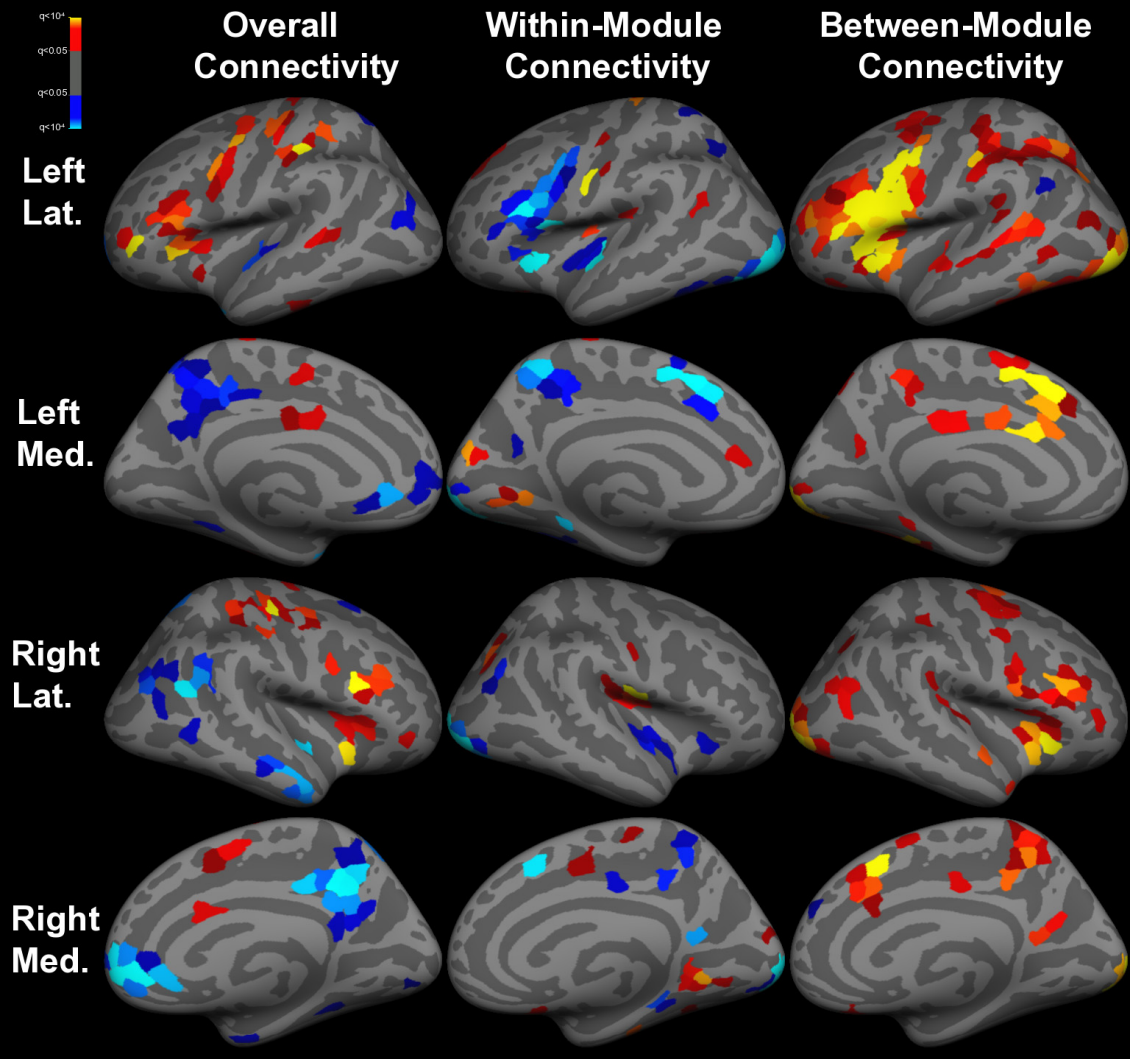
Task-related differences in functional connectivity. Inflated cortical surface representations of the significant (*q* < 0.05) differences in graph theoretical measures across 1000 ROIs between resting state and task runs. Warm colors indicate greater connectivity at rest, while cool colors indicate greater connectivity during task. Overall connectivity is degree, within-module connectivity is within-module z-score of degree, and between-module connectivity is participation coefficient. Light gray = gyri; dark gray = sulci.

Overall connectivity (Fig.3), as measured by degree, was greater among DMN areas as well as bilateral posterior insular and right anterior temporal cortices during the semantic decision task. On the other hand, overall connectivity was greater in bilateral primary sensorimotor, superior frontal gyri, inferior lateral frontoinsular, and left superior temporal cortices at rest.

### Within-module connectivity

Within-module connectivity (Fig.3) was increased among bilateral precunei, insulae, and superior frontal gyri as well as the left inferior frontal gyrus during the semantic decision task, while primary sensorimotor and visual cortices had greater within-module connectivity at rest.

### Between-module connectivity

With the exception of the dorsomedial left angular gyrus, which had greater between-module connectivity during semantic decision making, between-module connectivity (Fig.3) was diffusely greater at rest throughout the brain, especially in bilateral inferior frontal and insular cortices.

## Discussion

In this study, we employed a graph-based network approach to quantify changes in functional connectivity between the resting-state and during a semantic decision task. The principal finding was that task-related functional connectivity networks are more distinct, with a greater number of within-module connections and fewer between-module connections than during rest. We hypothesize that this reorganization in functional connectivity networks during a semantic decision task reflects the cortical computations involved including orthographic, lexical, phonetic, and semantic processing. The semantic processing may include attention effects as well as verbal memory.

### Default mode network

Although the DMN comprises cerebral areas with BOLD deactivations during task-related behaviors, this decrease in activity during memory-related task performance has been found to be associated with increased connectivity in these regions (Esposito et al. 2006, 2009; Hampson et al. 2006; Newton et al. 2011; Gordon et al. 2012; Li et al. 2012). This had led to the view that the DMN plays a role in higher level cognitive processing rather than representing an invariant physiological network or spontaneous activity (Hasson et al. 2009). However, a few studies have reported either no difference (Calhoun et al. 2008) or decreased (Buckner et al. 2009; Marrelec and Fransson 2011) intra-DMN connectivity during a cognitive task, suggesting a complicated relationship between task-dependent connectivity and cognitive function. The results of this study support the notion that multimodal areas of the DMN are indeed involved in semantic cognitive processes, but further work will be necessary to delineate these exact functions. Since greater intra-DMN connectivity has been associated with increasing cognitive load (Newton et al. 2011) and working memory performance (Hampson et al. 2006), we hypothesize that the increase in intra-DMN connectivity observed in this study during semantic decision making facilitates encoding and retrieval of verbal memory. This hypothesis is supported by task-based fMRI in epilepsy patients that show a semantic decision task can predict postoperative verbal memory deficits in patients who undergo a left temporal lobectomy (Binder et al. 2008).

In addition to exploring within-DMN connectivity, we also observed novel changes in internetwork connectivity involving the DMN, specifically decreased connectivity between DMN and language areas in the context of semantic decision making compared to rest. Although the DMN, especially its posterior subregions, has been identified as the principal connectivity hub in both structural (Hagmann et al. 2008) and resting-state functional connectivity analyses (Buckner et al. 2009), these results suggest that the DMN may exhibit a less hub-like pattern of connectivity during a task than at rest. These task-driven changes in network connectivity may provide a new metric by which the robustness of clinically relevant neural networks can be measured. It may provide a useful measure for localizing and lateralizing verbal function in patients, such as in the presurgical evaluation of epilepsy or brain tumor patients.

### Language network

Early fMRI studies employing a semantic decision task similar to that used in this experiment reported BOLD activations primarily in the left inferior frontal gyrus in healthy right-handed subjects (Demb et al. 1995; Gabrieli et al. 1996). In this study of right-handed healthy subjects, we observed a left-lateralized and highly intraconnected module involving classical language and cognitive control areas including bilateral opercular inferior frontal gyri, bilateral pre-SMA, the left posterior superior temporal gyrus, and the left angular gyrus (Niendam et al. 2012). Although bilateral inferior frontal areas had more overall connections at rest, more of those connections were intramodular (i.e., to other language areas) during the semantic decision task, suggesting that language processing is associated with an increase in within-network connectivity and a reduction in between-module connectivity. This supports our hypothesis that cognitive task-related changes in functional network organization tend toward highly intraconnected, less hub-dependent subnetworks.

While multiple models of the neural organization of the semantic system have been proposed, the findings of this study suggest that both multimodal hub areas of the DMN and classical language networks contribute to semantic processing. Alternatively, we observed that the dorsomedial left angular gyrus, which has been reported to be both activated during a broad range of semantic tasks and part of the DMN (Seghier et al. 2010), demonstrated increased between-module connectivity during semantic decision making, making it a putative connector hub between the DMN and language networks. This hypothesis would be supported by the existence of a previously proposed extra-arcuate indirect perisylvian white matter tract connecting both Broca and Wernicke's areas to the inferior parietal lobule (Catani et al. 2005). Additionally, increased posttherapy functional connectivity in posterior DMN areas, including the left angular gyrus, has been reported in patients undergoing treatment for aphasia suggesting that this region plays a role in integrating the functions of classical language and DMN regions (Marcotte et al. 2013).

### Resting-state networks as an idling state

As previously mentioned, many prior studies have demonstrated task-related reorganization of functional connectivity networks when compared to the resting state, leading to the hypothesis that resting-state networks represent an inactive state of a dynamic system. A proposed physiological mechanism of this change is task-related reduction in correlated noise, which has been observed at both the level of brain regions as well as in microcircuits (Smith and Kohn 2008; Nauhaus et al. 2009; Betti et al. 2013). The results of this study support the hypothesis that resting-state circuits represent an idle state which must be reorganized for task-related networks to emerge rather than a fixed prior whose connectivity is strengthened during task performance.

### Limitations

There are several factors that limit this study, some of which may merit future investigation. First, the acquisition voxel size and scan length differed between resting state and task runs. However, a prior study demonstrated that differences in voxel size do not affect measures of functional connectivity, and that functional connectivity was stabilized in runs as short as 5 min (Van Dijk et al. 2010). Second, although the entire cortex was studied using a surface-based approach, we cannot comment on potentially important changes in connectivity involving subcortical structures. Additionally, although we did not explore the relationship between task performance and changes in dynamic connectivity, future studies may address those questions.

## Conclusions

We observed task-related dynamics in functional connectivity that suggest that resting-state networks tend to be distributively connected, while functional connectivity subnetworks during semantic decision making are more distinct and intraconnected, especially DMN and language areas. We believe that these differences in network organization are related to language processing and semantic memory and represent a dynamic reconfiguration of an idling state. These changes may provide a useful means to measure the quality and dynamic range of functional connectivity networks in both clinical and research settings.
